# Photodynamic Microbial Defense of Cotton Fabric with 4-Amino-1,8-naphthalimide-Labeled PAMAM Dendrimer

**DOI:** 10.3390/ma18245570

**Published:** 2025-12-11

**Authors:** Desislava Staneva, Daniela Atanasova, Ivo Grabchev

**Affiliations:** 1 Department of Textile, Leather and Fuels, University of Chemical Technology and Metallurgy, 1797 Sofia, Bulgaria; d.atanasova1@abv.bg; 2 Faculty of Medicine, Sofia University “St. Kliment Ohridski”, 1407 Sofia, Bulgaria

**Keywords:** photoactive dendrimers, 1,8-naphtalimides, antibacterial textile, photodynamic inactivation, self-disinfected

## Abstract

The article describes the interaction between 4-amino-1,8-naphthalic anhydride and the terminal amine groups of the first-generation poly(amidoamine) (PAMAM) dendrimer. Cotton fabric was treated with the newly obtained photoactive dendrimer (DA) to achieve its antimicrobial photodynamic inactivation. The photodynamic inactivation method is an innovative approach in which, upon irradiation with visible light, photosensitizers generate highly reactive oxygen species, specifically singlet oxygen (^1^O_2_), which destroys microbial cells. In the dark, the DA dendrimer strongly inhibits the development of the model bacteria Bacillus cereus (a Gram-positive bacterium) and Pseudomonas aeruginosa (a Gram-negative bacterium) in solution. Upon irradiation with visible light, the inhibition is significantly enhanced, achieving almost complete inactivation of *B. cereus* and 94% of *P. aeruginosa*. Cotton fabric was treated with the DA dendrimer at two concentrations (0.15% and 0.30% weight of fabric). It was found that the dendrimer molecules are adherent to the cellulose fiber surfaces and do not leach in washing. Treatment of the fabric with DA partially increases its hydrophobicity, which prevents the adhesion of some bacteria. In the dark, the treated fabric shows weak antibacterial activity because the dendrimer DA molecules are attached to the textile surface, and inactivation depends solely on the microorganism’s surface contact. However, upon irradiation, a significant increase in the fabric’s antimicrobial activity is observed, as the fixed dendrimer participates in the release of singlet oxygen, which effectively attacks microorganism cell membranes and components. For the fabric with the higher concentration (DA30), 94% inactivation of *B. cereus* and 89% inactivation of *P. aeruginosa* were achieved. Thus, a synergistic effect between photodynamic activity and increased hydrophobicity was achieved, making the modified cotton fabric an example of a high-tech textile with permanent, renewable disinfection.

## 1. Introduction

**Scheme 1:**
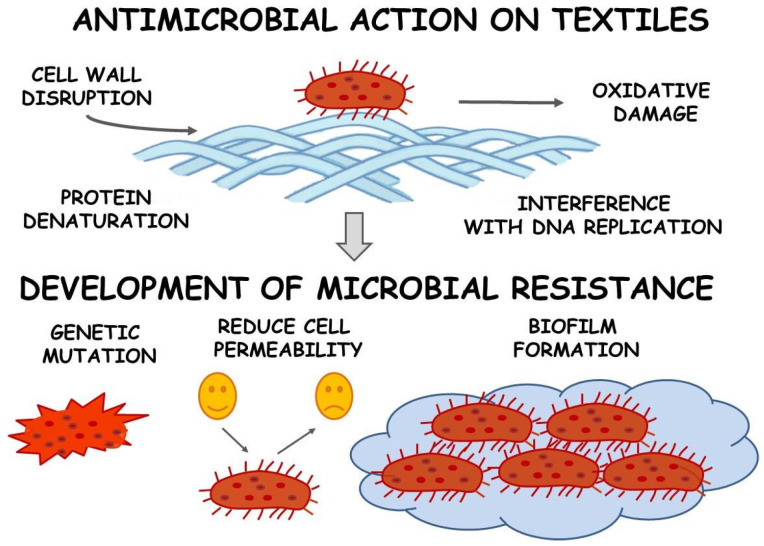
Antimicrobial action types on textiles and the ways for the development of microbial resistance.

Antimicrobial cotton fabric is a functional textile material in which the textile matrix is modified to inhibit the development of pathogenic microorganisms (bacteria, fungi, and viruses, etc.) [1]. It is increasingly used as a sanitary material, particularly for wound dressings, to aid healing and suppress the risk of nosocomial infections [2,3]. Antimicrobial textiles can be produced using various methods, including the application of nanoparticles and metallodendrimers [4,5,6,7,8], quaternary ammonium compounds [9,10], natural antibacterial agents [11,12], and low- and high-molecular biologically active substances [13,14,15], among others. Antimicrobial finishes on textiles can exhibit different properties. The active compounds can slowly release from the fabric surface and diffuse into the environment to interact with microbes, or they can be firmly bonded to the fibers and affect only the microorganisms that encounter the fabric surface. They can damage or break the microbial cell wall or membrane, leading to the leakage of cell contents and cell death. Alternatively, they can enter the cell and destroy metabolic pathways or inhibit DNA synthesis. Additionally, they can interact with cellular proteins, enzymes, or nucleic acids, leading to inactivation or denaturation. They can also promote the formation of reactive oxygen species, which damage lipids, DNA, and proteins. The different ways in which textile materials affect bacteria, leading to their inactivation, are summarized in Scheme 1.

Over the past few years, our laboratory has carried out extensive research on the synthesis of new antimicrobial agents on the basis of 1,8-naphthalimide (NI) and their use in the treatment of textile materials [15,16]. 1,8-Naphthalimide is a polycyclic aromatic compound with a planar structure that enables it to intercalate between DNA base pairs, thereby disrupting DNA replication and transcription in bacteria [17,18,19,20]. This intercalation is a crucial mechanism of antimicrobial action, as it prevents enzymes from reading or replicating genetic material. The substituents strongly influence the antibacterial activity of 1,8-naphthalimide derivatives, as electron-accepting groups enhance interaction with bacterial enzymes. In contrast, electron-donating groups improve water solubility and enhance hydrogen bonding with DNA or proteins. In the presence of amphiphilic properties, 1,8-naphthalimide can be incorporated into the bacterial membrane, disrupt its structure and permeability, cause leakage of cellular contents, and cell death [21].

To increase the activity of 1,8-naphthalimide as an antimicrobial agent, high-molecular substances, including dendrimers, were modified with it. Dendrimers with positively charged ends, such as poly(amidoamine) or poly(propylenimine), in which 1,8-naphthalimides are attached to the periphery of the dendrimer, have been used. The functional hybrid materials are prepared in this way, combining the stability and multifunctionality of dendrimers with the excellent biological activity of NI [22]. On the other hand, dendrimers often increase the solubility of 1,8-naphthalimides in both aqueous and organic media, thereby reducing the probability of aggregation and thus controlling the size of the resulting nanostructures. Additionally, due to their intense fluorescence, they are utilized as bright, photostable fluorescent probes for monitoring processes in living cells [23] or as highly sensitive fluorescent sensors for pH monitoring and the detection of metal ions and anions [24,25].

Some 1,8-naphthalimide-modified dendrimers exhibit well-expressed antimicrobial and antitumor activity [26,27,28]. They can destabilize the lipid bilayer and disrupt membranes, especially of Gram-negative bacteria, via electrostatic attraction to their negatively charged surfaces. As a result, the attached 1,8-naphthalimide groups then more easily enter the cell and reach DNA or enzyme targets [29]. The dendrimers have a multivalent effect arising from the increased local concentration of 1,8-naphthalimide units around the bacterial cell, which is key to their antibacterial activity.

However, since the beginning of the 21st century, antibiotics used in clinical practice have gradually lost effectiveness due to the emergence of pathogenic microorganisms resistant to them [30,31,32,33]. The rapid microbial reproduction contributes to their adaptability and, as a result, the infections they cause are among the most common causes of mortality. The adaptability of microorganisms, due to their rapid reproduction, contributes to their increasing resistance to antimicrobial agents, making this type of infection one of the most serious causes of mortality worldwide [34,35,36,37]. Over time, microorganisms can also adapt to textile antimicrobial finishes through several mechanisms. They can alter their active sites by mutation, reduce cell permeability, and actively expel antimicrobial agents. Biofilm formation and the release of enzymes that degrade antimicrobial agents can also protect them (Scheme 1). They can be adapted and resistance to other antimicrobials can be built with similar mechanisms.

For this reason, the spread of resistant, especially multi-resistant, microorganisms represents a significant challenge for treating the diseases they cause. Therefore, the development and introduction of new, highly effective substances and methods into clinical practice are urgently needed to regain protection from pathogenic microorganisms [38,39].

In this regard, new compounds called photosensitizers (PSs) are designed and synthesized. Their role in antimicrobial photodynamic inactivation (APDI) is currently being intensively investigated as an innovative approach and a promising strategy for inactivating a wide range of pathogenic microorganisms, including bacteria, fungi, viruses, and protozoa [40,41,42,43,44]. APDI is based on the generation of highly reactive oxygen species (ROS), including singlet oxygen (^1^O_2_), which attack and destroy microbial cells [45,46,47,48]. Recently, special attention has been paid to the use of photosensitizers deposited on textile surfaces. With this addition, the fabric becomes a heterogeneous system that generates ROS and ^1^O_2_ upon exposure to light. These species attack the cell walls, membranes, and DNA of microorganisms that come into direct contact with the fabric surface [49,50]. The mechanism of action is rapid, non-thermal, and highly effective against resistant strains because destruction occurs via physicochemical processes rather than specific metabolic pathways. The hydroxyl groups in cotton fabric facilitate stable attachment of photosensitizers through covalent, ionic, or hydrogen bonds. This stability helps maintain the high efficiency of ^1^O_2_ generation in practical applications of functional textiles.

In recent years, 1,8-naphthalimide derivatives (NIs) have been investigated as photosensitizers that generate mainly singlet oxygen (^1^O_2_) via a type II photochemical mechanism [49,50,51]. These fluorophores are particularly attractive photosensitizers, since they have an easily functionalizable structure, especially at position C-4 of the 1,8-naphthalene ring and the imide nitrogen. To convert 1,8-naphthalimides from highly fluorescent compounds into efficient generators of singlet oxygen, the precise design of their photophysical properties is necessary to facilitate the intersystem crossing (ISC) process. The factors that increase spin–orbit coupling in the molecule facilitate the transition between the excited singlet (S1) to the excited triplet (T1) state, which is a prerequisite for the generation of ^1^O_2_ [52,53]. The ISC rate can be increased and thus enhance singlet oxygen generation by introducing substituents with weak electron-donating properties, heavy atoms and halogens. The transition from C=O to C=S, or the incorporation of sulfur into the structure of 1,8-naphthalimides, alters the energy levels of the excited states. In particular, a new nπ* triplet state can be introduced, which facilitates ISC, since the spin–orbit coupling is stronger between states with different orbital character (ππ→nπ*) [54].

This study aims to synthesize a new photoactive dendrimer by modification of the terminal amine groups of the first-generation poly(amidoamine) (PAMAM) dendrimer with 4-amino-1,8-naphthalic anhydride. The influence of organic solvent polarity on the photophysical properties of the dendrimer and its activity against model Gram-positive and Gram-negative pathogenic bacteria is assessed in the dark and under visible-light illumination. Cotton fabric was treated with the newly synthesized photoactive dendrimer, and its antibacterial activity against the same bacterial strains was evaluated to produce antibacterial, self-disinfecting cotton fabrics.

## 2. Materials and Methods

The methods, chemicals, and apparatus used in this work are presented in the Supplementary Materials.

### Synthesis of 4-Amino-1,8-naphthalimide-Labeled Poly(Amidoamine) Dendrimer (DA)

Poly(amidoamine) dendrimer from first-generation (0.143 g, 0.1 mmol) and 4-amino-1,8-naphthalic anhydride (0.133 g, 0.8 mmol) were dissolved in 50 mL of ethanol and stirred for 3 h at 60 °C. The reaction was monitored using thin-layer chromatography (TLC). After cooling, the liquor was poured into 300 mL of alkali ice water, and the resulting precipitate was filtered and dried in a vacuum. Yield: 0.281 g (94.0%).

FTIR, cm^−1^: 3352, 3312, 3074, 2944, 2827, 1630, 1572, 1366, 1244, 1056, 831, 754, 722.

1H NMR (DMSO_d6_) δ ppm: 8.53 (bs, 8H, Ar–H), 8.33 (s, 8H, Ar–H), 8.11 (s, 8H, Ar–H), 7.95 (s, 8H, NHCO), 7.74 (s,4H, NHCO), 7.55 (s, 8H, Ar–H) 7.36(s.16H, NH2), 6.78 (bs, 8H, Ar–H), 4.05 (s, 16H, CONH*CH*_2_), 3.02 (s, 16H, CONHCH_2_CH_2_) 3.01 (bs. 16H, NCH_2_CH_2_), 2.64 (bs, 24H, CONH_CH2_), 2.18 (s, 8H, CH_2_CO), 2.07 (bs, 16H), *CH*_2_CO), 1.24 (s 4H, >N*CH*_2_*CH*_2_N<); 13C NMR (DMSO_d6_) δ ppm: 171.8, 164.4. 162,5, 163.5, 153.0, 134.2, 131.3, 130.3, 129.6, 124.3, 122.4, 119.7, 108.5, 108.1, 52.5, 50.16, 49.9, 38.69, 37.1, 33.6.

Analysis: C_158_H_168_N_34_O_28_ (2991.25 g mol^−1^): Calc. (%): C-63.88, H 5.59, N 15.90; Found (%): C-63.83, H 5.44, N 15.74.

## 3. Results and Discussion

### 3.1. Synthesis of Fluorescent Dendrimer DA

**Scheme 2:**
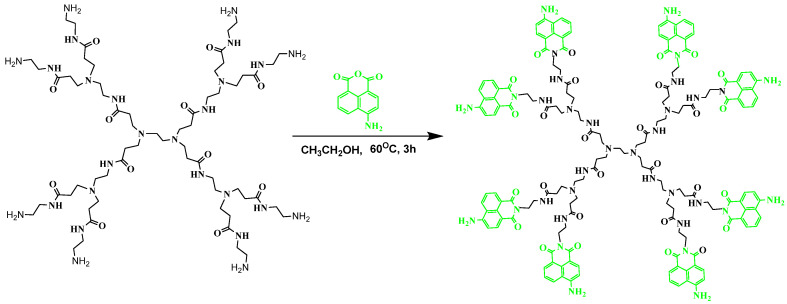
Synthesis of dendrimer DA.

A first-generation poly(amidoamine) dendrimer, which has eight primary amino groups, reacts with 4-amino-1,8-naphthalic anhydride (NA), in ethanol solution at 60 °C (Scheme 2). Thin-layer chromatography showed that 1,8-naphthalic anhydride was completely consumed within 3 h. The final product, dendrimer DA, dissolved in alcohol, was precipitated by pouring onto ice. Then the product was separated from the solution by filtration, washed with water, and air-dried. Infrared spectroscopy, 1H and 13C NMR, and elemental analysis were used to confirm and characterize the chemical structure of the dendrimer.

### 3.2. FTIR Spectral Characterization

**Figure 1:**
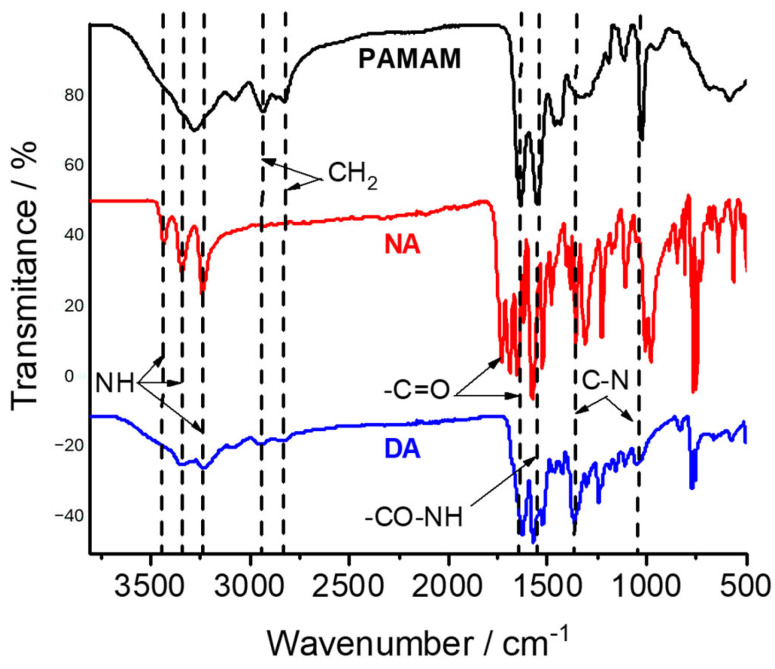
FTIR spectra of PAMAM, NA, and dendrimer DA.

Infrared spectroscopy has been used to characterize 1,8-naphthalimides and their derivatives, including dendrimer structures modified with them. This technique provides valuable information about the presence of their functional groups. Figure 1 compares the spectra of the starting 4-amino-1,8-naphthalic anhydride (NA), PAMAM dendrimer, and the dendrimer DA obtained from their interaction.

The main bands in the infrared spectrum of the PAMAM dendrimer arise from the vibrations of the main functional groups that make up its structure: amide groups (C=O, N-H), primary amine (–NH_2_), methylene groups (–CH_2_−), and tertiary amino groups (–N<) from the core of the dendrimer structure. The stretching of the N-H bonds of the terminal primary amino groups is recorded as two peaks at 3283 and 3351 cm^−1^. At 1636 cm^−1^, a strong band of C=O stretching of the Amide I (CONH) was observed, while the band for Amide II is at 1552 cm^−1^, which indicates the presence of amide bonds in the dendrimer structure. The stretching of the C-H of the methylene groups (-CH_2_−) is at 2938 and 2825 cm^−1^, and the characteristic deformation vibrations are at 1462 cm^−1^. The bands at 1026 and 1116 cm^−1^ are attributed to C-N vibrations of the tertiary amino groups in the dendrimer structure.

The most characteristic and recognizable bands in the IR spectra of 1,8-naphthalimide derivatives are due to symmetric and asymmetric vibrations of C=O groups. In the structure of NA, the two bands are at 1726 cm^−1^, ν^as^_C=O_, and 1685 cm^−1^, for ν^s^_C=O_. In DA, due to the formed imide structure, they shift to shorter frequencies. The asymmetric stretching (ν^as^_C=O_) is observed as a shoulder at 1680 cm^−1^, since it is strongly suppressed by the amide (HNC=O) groups of the dendrimer structure, while the symmetric stretching (ν^s^_C=O_) has an intense band at 1628 cm^−1^. This shift provides evidence for the formation of the 1,8-naphthalimide structure upon interaction of NA with the primary amino groups of PAMAM dendrimer.

As a substituent at the C-4 atom of the naphthalene structure of NA, there is a primary amino group, whose characteristic vibrational bands for N-H are registered as a triplet at 3432, 3337, and 3234 cm^−1^. In the dendrimer DA, they are registered as a well-pronounced doublet at 3354 and 3223 cm^−1^. In the structure of the dendrimer DA, there are two types of C-H bonds: aromatic and aliphatic. The characteristic stretching bands of the aromatic C-H bonds are of weak intensity around 3074 cm^1^ and bands of stronger intensity at 754 and 722 cm^−1^, which are absent in the spectrum of the PAMAM dendrimer. In the aliphatic C-H bonds, two bands characteristic of asymmetric stretching at 2944 cm^−1^ and symmetric stretching at 2827 cm^−1^ were observed. These signals are absent in the NA spectrum. The aromatic ring vibrations at DA (C=C stretching) are at 1572 and 754 cm^−1^. The stretching frequencies of the C-N bonds of the imide ring (O=C-NR-C=O) are at 1158 cm^−1^ and 1056 cm^−1^. The bands at 1366 and 1244 cm^−1^ are also characteristic of C-N bonds.

### 3.3. SEM Analysis of 4-Amino-1,8-naphthalic Anhydride and Dendrimer DA

**Figure 2:**
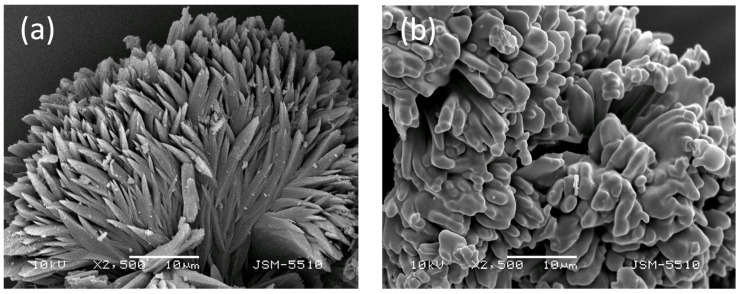
SEM micrographs of NA (**a**) and DA (**b**) at magnification ×2500.

In Figure 2a, the SEM micrograph of NA shows its radially organized crystalline structure. The individual units are thin and elongated, resembling needles, which appear to grow outward from a central point, forming a flower-like or rosette arrangement. In contrast, Figure 2b shows that the dendrimer DA has a much more granular and agglomerated, cauliflower-like morphology. Its construction is dominated by smooth, rounded, amorphous, irregularly shaped structures that adhere to one another and form small cavities. They create a compact and dense cluster with a globular growth pattern.

### 3.4. Photophysical Characteristics of the Dendrimer DA

Table 1 shows the photophysical characteristics of the dendrimer DA in organic solvents of different polarity.

**Figure 3:**
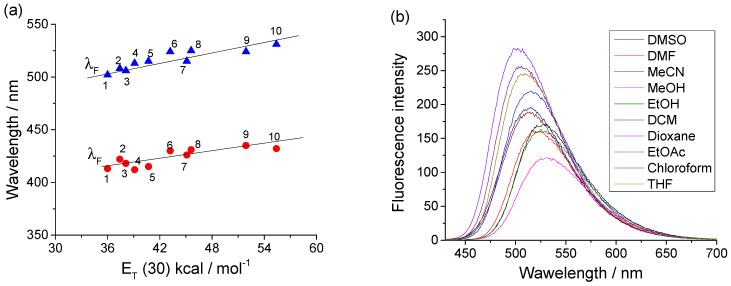
Dependence of absorption and fluorescence maxima (**a**) of dendrimer DA on the empirical parameter of solvent polarity E_T_(30): 1—dioxane, 2—tetrahydrofuran, 3—diethyl acetate, 4—chloroform, 5—dichloromethane, 6—DMF; 7—acetonitrile, 8—dimetyl sulfoxide, 9—ethanol, 10—methanol, and fluorescence spectra of dendrimer DA in organic solvents (**b**).

In the eight organic solvents studied, DA exhibits a wide range of absorption maxima (λ_A_) from 412 nm (chloroform) to 435 nm (ethanol), indicating that the dendrimer is highly sensitive to the polarity of the organic solvents. When comparing the λ_A_ values with the polarity of the solvent (Figure 3a), a trend is observed in which the absorption maximum shifts to longer wavelengths (bathochromic shift) in more polar protic solvents (methanol: 432 nm, ethanol: 435 nm) compared to less polar or moderately polar aprotic solvents (dichloromethane: 415 nm, dioxane: 413 nm, chloroform: 412 nm). Highly polar aprotic solvents such as dimethyl sulfoxide, *N*,*N*-dimethylformamide, and acetonitrile fall in an intermediate range (426–431 nm).

The observed bathochromic shift in λ_A_ with increasing solvent polarity, especially in protic solvents, implies that the 1,8-naphthalimide fragments of the dendrimer structure undergo a π→π* electronic transition, in which the excited state is significantly more polar and/or more stabilized by its interactions with the solvent (e.g., hydrogen bonding) than its ground state. This increased stabilization of the excited state by polar solvents effectively reduces the energy difference between the ground and excited states, leading to the absorption of lower-energy (longer-wavelength) photons.

Fluorescence emission maximum (λ_F_) ranges from 502 nm (dioxane) to 531 nm (methanol). Similarly to the absorption maximum, λ_F_ also exhibits a significant red shift in more polar solvents (Figure 3b). For example, methanol (531 nm) and ethanol (524 nm) exhibit longer emission wavelengths compared to dioxane (502 nm) and ethyl acetate (506 nm). The pronounced bathochromic shift in the position of λ_F_ with increasing solvent polarity further supports the conclusion that the excited state of the compound is significantly more polar than its ground state.

Upon excitation, the dipole moment of the molecule changes, leading to a reorientation of the surrounding solvent molecules and stabilization of the new charge distribution in the excited state (solvent reorganization). This process lowers the energy of the excited state before emission, resulting in photons with lower energy.

**Figure 4:**
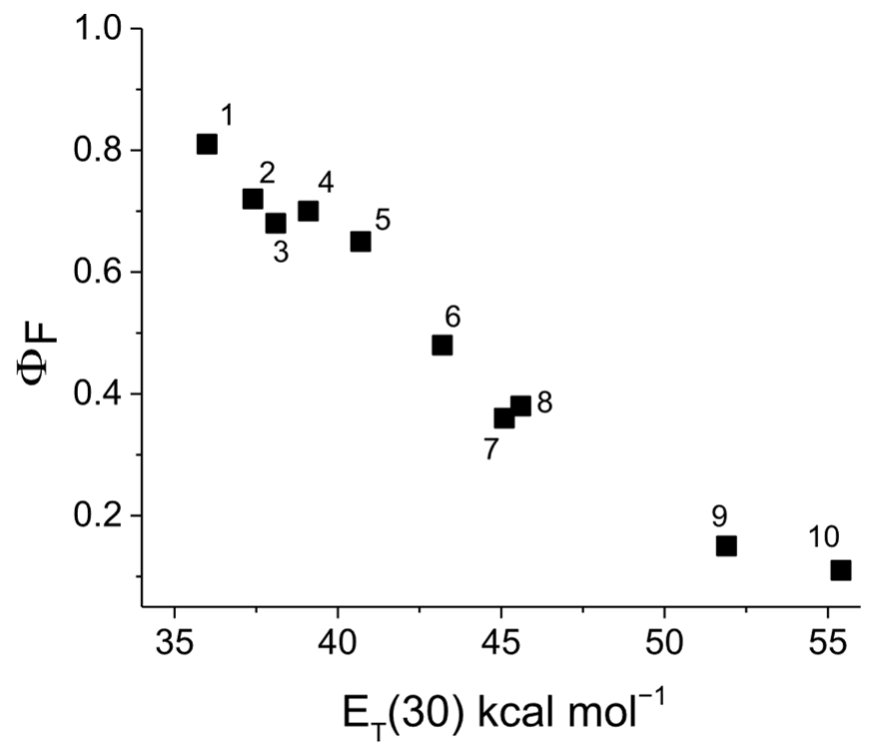
The influence of solvent polarity empirical parameter E_T_(30) on the dendrimer DA fluorescence quantum yield: 1—dioxan; 2—tetrahydrofuran; 3—diethyl acetate; 4—chloroform; 5—dichloromethane; 6—DMF; 7—acetonitrile; 8—dimetyl sulfoxide; 9—ethanol; 10—methanol.

The fluorescence quantum yield (Φ_F_) also depends on the type of solvents and ranges from high values of Φ_F_ = 0.81 (dioxane) and Φ_F_ = 0.72 (tetrahydrofuran) to lower values of Φ_F_ = 0.11 (ethanol) and Φ_F_ = 0.15 (methanol) (Figure 4). A significant decrease in Φ_F_ in polar protic solvents (methanol and ethanol) compared to nonpolar or moderately polar aprotic solvents (dioxane, tetrahydrofuran, chloroform, and dichloromethane) was observed. This indicates that nonradiative deactivation pathways become much more efficient in protic solvents. As a result, fluorescence is effectively quenched. This also increases the potential for Intersystem Transition (ISC) from the singlet excited state (S1) to the triplet excited state (T1).

Lower quantum yields in less viscous, protic solvents may be related to increased molecular flexibility. Excited-state conformational changes may also favor nonradiative energy dissipation. Conversely, higher Φ_F_ in more viscous or less interacting media (dioxane, tetrahydrofuran) suggests that the molecule maintains a more favorable emission conformation. It may also undergo fewer nonradiative transitions.

The Stokes shift is a quantity that characterizes the properties of fluorophores in their normal and first excited states and is calculated as the difference between the positions of the absorption and fluorescence maxima. From the data in Table 1, it can be seen that the Stokes shift is in the range 3996–4973 cm^−1^, and these values also depend on the solvent polarity, as they yield higher values in nonpolar solvents.

The molar extinction coefficients (ε) for DA are high in all solvents tested, ranging from 79,780 L mol^−1^ cm^−1^ (tetrahydrofuran) to 93,500 L mol^−1^ cm^−1^ (dichloromethane), which is approximately 8 times higher than the monomeric 4-aminosubstituted-1,8-naphthalimide [24,25,55]. These high ε values indicate that the dendrimer is a robust light absorber across the solvents, suggesting a well-resolved electronic transition with a significant transition dipole moment, making it an effective chromophore.

### 3.5. Treatment of Cotton Fabrics with Dendrimer DA

Antimicrobial textile materials can neutralize or destroy pathogenic microorganisms (bacteria, fungi, viruses, etc.), thereby acquiring self-disinfection properties. This special textile finishing is achieved by using different antimicrobial agents or by applying coatings that can react with light or other factors to neutralize microorganisms on the textile surface [56]. Low-molecular-weight compounds are usually readily soluble and bind weakly to textile fibers via physical adsorption, leading to rapid removal from the textile surface, whereas dendrimers, due to their low water solubility and large number of functional groups, promote permanent attachment. On the other hand, monomeric antimicrobial agents have only one active site to target bacterial cells. Dendrimers, due to their highly branched structure, have many times more active groups and, through their multivalent effect, simultaneously engage multiple interactions with the bacterial cell, significantly increasing the probability and speed of microorganism destruction.

For this purpose, cotton fabric was treated with two ethanol solutions of dendrimer DA at concentrations of 0.15% (DA15) and 0.30% (DA30) by weight of fabric, at 50 °C for 60 min. At this concentration, the amount of dendrimer deposited on 1 cm^2^ is equivalent to the amount of the studied dendrimer in solution, allowing comparison of its antimicrobial activity in the two environments. After treatment, the fabric was dried in the air, then washed first with a detergent solution at room temperature, then with water, and finally dried again. The dendrimer molecule has a high molecular weight and contains multiple functional groups capable of interacting with hydrogen and Van der Waals bonds to the cellulose macromolecules of the fabric. The dendrimer DA is insoluble in water, further stabilizing it on the cotton surface and preventing easy detachment.

Table 2 presents the coordinates for untreated cotton fabric DA, along with those for cotton fabrics treated with 0.15% wof (DA15) and 0.30% wof (DA30). The parameter L* represents the lightness of the cotton material and decreases after treatment due to the presence of the dendrimer on the fabric surface. Negative values of the parameter a* indicate a shift towards green, while positive values of the parameter b* reflect a shift towards yellow. The dendrimer used in this study has a yellow hue with intense yellow-green fluorescence, which contributes to the observed changes in the parameters L*, a* and b* of the treated cotton fabrics. Furthermore, the color slightly intensifies with increasing DA concentration (ΔE* = 40.68 for DA15 and 42.96 for DA30, respectively). On the other hand, it was observed that after washing with the detergent, the changes in the color parameters L*, a*, b* and ΔE* were insignificant, indicating that the dendrimer is firmly attached to the cotton surface and does not release into solution.

### 3.6. Hydrophilicity of Cotton Fabrics

Humidity is one of the most critical factors influencing the spread of pathogenic microorganisms on cotton fabrics. Humidity is one of the most critical factors influencing the spread of pathogenic microorganisms on cotton fabrics. Increased humidity, both in the fabric itself and in the ambient air, provides the necessary water for metabolic processes and the reproduction of microorganisms. Cotton is hygroscopic and absorbs moisture. High humidity, combined with heat and nutrients from human body waste, such as sweat, oil, and laundry residues, also promotes the growth of various types of pathogenic bacteria that cause diseases or unpleasant odors. Microorganisms can form biofilms on moist surfaces, making them more resistant to cleaning and disinfection.

To assess the hydrophobicity of DA-treated cotton fabric, the samples of DA15 and DA30 were immersed in deionized water for 5 min. The following formula was used to determine the amount of adsorbed water [57]:

*Adsorption (%) = (mass of adsorbed water/initial mass) × 100.*

The untreated cotton fabric adsorbed 114% of the water. The water absorption of the treated cotton fabrics was less pronounced: with DA15, 91% was retained, and with DA30, 83% was retained. The results showed that the DA dendrimer increased the fabric’s hydrophobicity by interacting with its hydroxyl groups.

### 3.7. Reactive Oxygen Species (^1^O_2_) Generated from Dendrimer DA and Treated Cotton Fabric with It

The singlet oxygen (^1^O_2_) generation from dendrimer DA at a concentration of c = 1 × 10^−6^ M in aqueous solutions, or from cotton fabrics with DA15 and with DA30 immersed in water, was studied by the iodometric method upon irradiation with visible light.

In this case, 4-amino-1,8-naphthalimides (DA-NI) fragments of the dendrimer structure were used as photosensitizers due to their photophysical characteristics, which enable them to efficiently generate singlet oxygen (^1^O_2_) through a photosensitized mechanism (Type II reaction) upon irradiation with light [58,59].

Light absorption: The 1,8-naphthalimide (DA-NI) molecule absorbs a photon and goes to an excited singlet state (^1^DA-NI*):

DA-NI + sunlight→^1^DA-NI*

Intersystem crossing (ISC): ^1^DA-NI* goes to an excited triplet state (^3^DA-NI*), since 1,8-naphthalimides often have a high ISC rate:

^1^DA-NI*→^3^DA-NI*

Energy transfer: ^3^DA-NI* transfers energy to molecular oxygen in the ground triplet state (^3^O_2_), generating singlet oxygen (^1^O_2_), and ^3^DA-NI* returns to the ground state

^3^DA-NI^*^ + ^3^O_2_→DA-NI + ^1^O_2_

The triiodide anion is formed when the iodide ion (I^−^) reacts with an iodine molecule (I_2_) in a reversible equilibrium reaction:

I^−^ + ^1^O_2_ → IOO^−^ → IOOH

IOOH + I^−^ → IOOH_2_^−^ → I_2_ + OH_2_^−^ → I_3_^−^ + OH^−^ + H_2_O_2_

**Figure 5:**
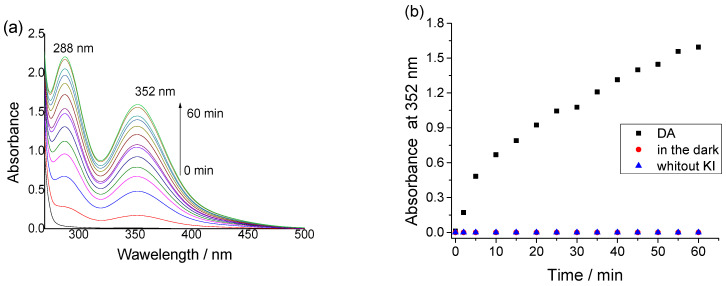
Absorption spectra of I_3_^−^ in the presence of dendrimer DA upon irradiation (**a**) and influence of the time on the absorbance value at 352 nm: without KI, with DA in the dark, and with DA with irradiation (**b**).

**Figure 6:**
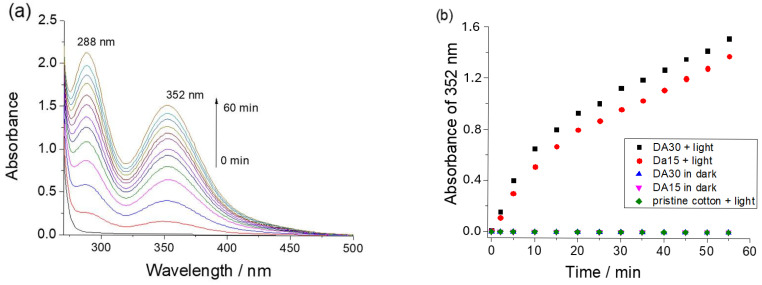
Absorption spectra of I_3_^−^ treated with dendrimer DA cotton fabric upon irradiation (**a**) and influence of the time on the absorbance value at 352 nm of pristine cotton with irradiation, with DA15 and DA30 in the dark, and with DA15 and DA30 with irradiation (**b**).

The absorption spectra of I_3_^−^ in the presence of dendrimer DA upon irradiation were obtained within the spectral range 270–500 nm and are presented in Figure 5a. The data show that the unirradiated KI solution does not show absorption in this range. However, after irradiation, two distinct absorption maxima appear at 288 nm and 352 nm, which are characteristic of I_3_^−^ ions formed by the photooxidation of I^−^ by the singlet oxygen (^1^O_2_) generated during irradiation of dendrimer DA. At the studied dendrimer concentration, aggregation processes are expected, which would reduce the amount of ^1^O_2_. Cotton fabrics processed with dendrimer were also tested for singlet oxygen generation in the dendrimer solution. The results for the cotton fabric treated with DA30 are shown in Figure 6a. The absorption spectra are similar to those of dendrimer DA, indicating that it retains its ability to generate singlet oxygen after being immobilized on the fabric surface. The dendrimer is retained on the cotton fabric through multiple hydrogen bonds with cellulose hydroxyl groups. In this way, the 1,8-naphthalimide fragments of DA are physically separated and isolated from one another, thereby preventing aggregation. The hydrogen bonds probably further stabilize the dendrimer molecule in a specific conformation. This conformation is more favorable for the generation of singlet oxygen. It was also observed that increasing the amount of dendrimer on the cotton fabric surface led to greater production of reactive oxygen species (Figure 6b). The absorbance of the dark and irradiated solution without KI was measured. No absorbance was detected in the studied spectral region. No absorption was also recorded in the KI solution in the presence of untreated cotton fabric. These results clearly indicate that singlet oxygen is not generated under these conditions.

### 3.8. Antimicrobial Effect of Dendrimer DA and Cotton Fabrics DA15 and DA30

**Figure 7:**
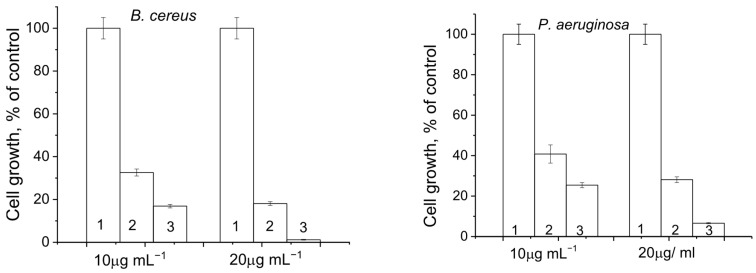
The growth of the model bacteria *B. cereus* and *P. aeruginosa* in the presence of the dendrimer DA (at concentrations of 10 µg mL^−1^ and 20 µg mL^−1^): 1—control; 2—in the dark, and 3—after light illumination.

**Figure 8:**
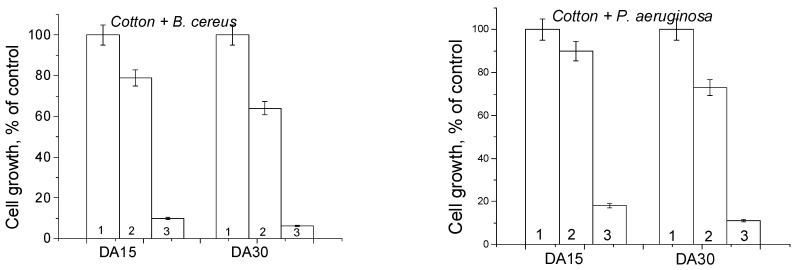
The growth of the bacteria *B. cereus* and *P. aeruginosa* in the presence of a treated fabric with dendrimer DA: 1—control; 2—in the dark; 3—under visible light.

The antimicrobial activity of the dendrimer DA and its response to light were evaluated against two bacterial strains: *B. cereus* (a Gram-positive bacterium) and *P. aeruginosa* (a Gram-negative bacterium). Tests were performed using DA solutions at 10 µg mL^−1^ and 20 µg mL^−1^ (Figure 7) and on cotton fabrics DA15 and DA30 (Figure 8).

The results obtained showed that DA solutions inhibited the growth of the model cultures compared to the negative control. In the dark, the activity of DA was better expressed on *B. cereus*. At a concentration of 10 µg mL^−1^ in the dark, DA reduced the growth of *B. cereus* by about 68%, while for *P. aeruginosa*, it reduced the growth by about 59% compared to the control, set at 100%. At twice the amount of dendrimer, the inactivation was 82% and 72%, respectively. In this case, the inhibition of bacterial growth is due to the direct interaction of DA with bacterial membranes, leading to changes in their membrane characteristics, such as lipid area, monolayer elasticity, lipid molecular organization, and membrane order [29].

After irradiation with light, due to the generation of singlet oxygen, inhibition of bacterial growth increased to about 83% (*B. cereus*) and 75% (*P. aeruginosa*), respectively. At double the concentration (20 μg mL^−1^), light irradiation almost completely inhibited the growth of *B. Cereus*, and the inhibition of *P. aeruginosa* was 94% (Figure 7).

These observations suggest a significant enhancement of the antimicrobial effect under light irradiation. This effect is attributed to the dendrimer’s generation of singlet oxygen, which attacks bacterial cells, causing severe oxidative damage to key cellular components. The resulting damage occurs through oxidative reactions, with primary targets in the bacterial cell including the cytoplasmic membrane, proteins and enzymes, nucleic acids, and unsaturated fatty acids. This leads to lipid peroxidation, disrupts membrane integrity and function, and ultimately results in bacterial damage and death [60,61].

Gram-positive bacteria are susceptible to ^1^O_2_ attack because their peptidoglycan layer is dense, but its porous structure facilitates the penetration of the photosensitizer and the action of singlet oxygen. In comparison, an additional outer membrane in Gram-negative bacteria protects them from easier penetration.

The antimicrobial activity of textile fabrics DA15 and DA30 was investigated after immersion in MPB solution containing the same model bacterial strains as for the DA dendrimer, both in the dark and after irradiation. The results show that the inhibition of bacterial growth follows the trend observed for the DA dendrimer (Figure 6). This means its activity is maintained after it is deposited on the cotton fabric. In the dark, cotton fabrics exhibit significantly lower antibacterial activity than the dendrimer. In DA15, the inhibition against *B. cereus* is 27%; against *P. aeruginosa*, 10%; and against DA30, 36% and 27%. This low activity can be attributed to the fact that the dendrimer molecules are attached to the surface of the cotton matrix and are not released into the solution, thereby limiting their contact with microorganisms that are only in proximity to the fabric.

Upon irradiation, a significant enhancement of antimicrobial activity against both pathogenic strains was observed, with *B. cereus* inactivation reaching 94% and *P. aeruginosa* inactivation reaching 89% (DA30 fabric), due to the ^1^O_2_ generated by the dendrimer deposited on the fabric. In this way, the bacteria are prevented from remaining on the fabric and forming colonies and biofilms, which are crucial for creating self-sterilizing textiles (Figure 8).

To assess the long-term effects, cotton fabrics were tested after their 10th wash. The results showed only a slight decrease of 4–8% in activity compared to the initial values, indicating good long-term performance.

### 3.9. SEM Investigation of Cotton Fabrics

**Figure 9:**
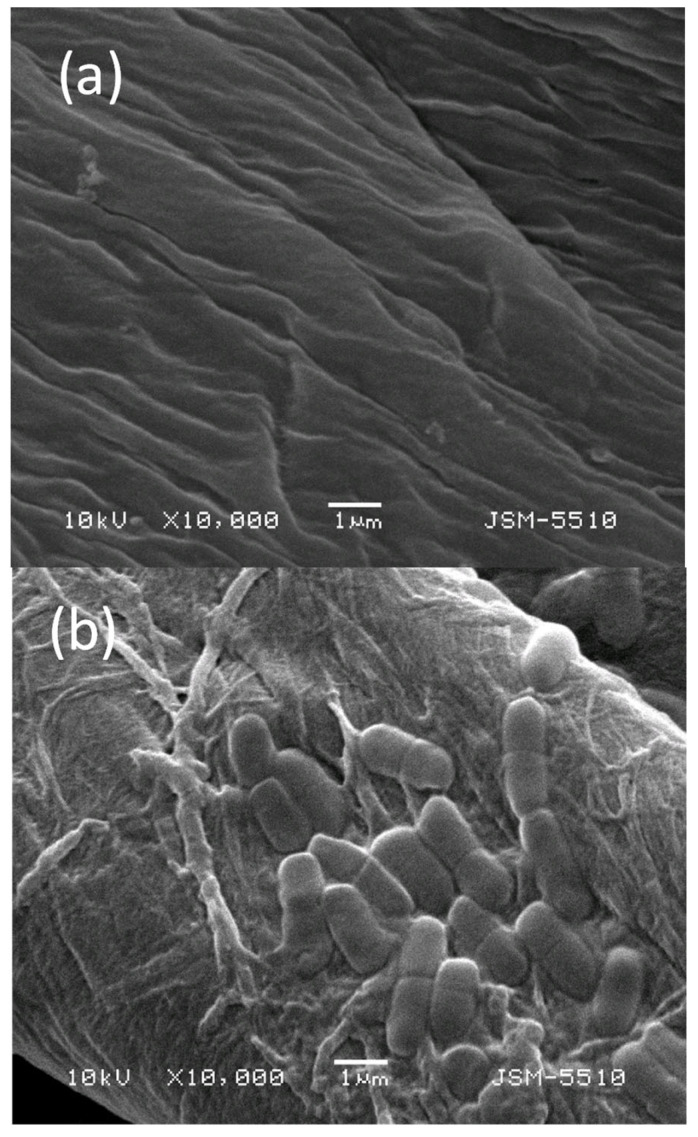

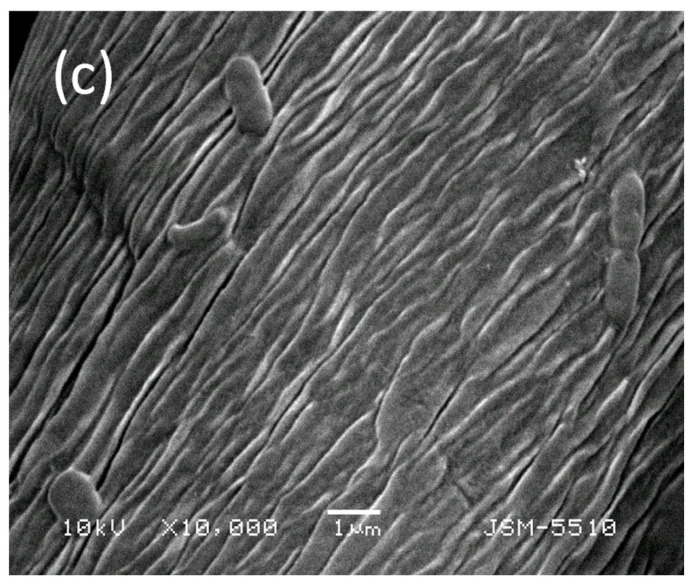
SEM micrographs of pristine cotton fabric (**a**); untreated cotton fabric with biofilm of *P. aeruginosa* (**b**); DA30-treated cotton fabric in contact with *P. aeruginosa* under irradiation (**c**) at ×10,000 magnification.

Scanning electron microscopy (SEM) was used to examine the cotton surfaces of dendrimer DA-treated and untreated fabrics for bacterial attachment and biofilm formation. Figure 7 compares SEM images of pristine cotton fabric (A); untreated cotton fabric in contact with *P. aeruginosa* (B); and DA30-treated cotton fabric in contact with *P. aeruginosa* under irradiation (C). Figure 9a shows the classic fibrillar structure of untreated cotton. After contact with bacteria, a stable *P. aeruginosa* biofilm formed on the cotton, supported by a bacterial matrix (Figure 9b). In contrast, after DA treatment, no biofilm appeared. Only single bacterial cells were seen on the cotton (Figure 9c). Dendrimer-treated fabric is more hydrophobic than untreated cotton, thereby hindering bacterial adhesion. The dendrimer also generates singlet oxygen under irradiation, directly on the surface of the cotton fabric, which attacks and destroys bacteria that attach to the cotton. This results in a synergistic effect, giving the cotton antibacterial and self-disinfecting properties.

## 4. Conclusions

In conclusion, a first-generation fluorescent PAMAM dendrimer modified with eight 4-amino-1,8-naphthalimide units (DA) was synthesized and characterized. Upon visible light irradiation, the dendrimer generated reactive singlet oxygen via a photosensitized mechanism (type II reaction) using its 4-amino-1,8-naphthalimide moieties as photosensitizers, which underlies its antimicrobial photodynamic activity. In a 20 µg mL−1 solution in the dark, the dendrimer inhibited the growth of the model bacterial strains *B. cereus* (Gram-positive) and *P. aeruginosa* (Gram-negative) by 82% and 72%, respectively. A significant enhancement of its activity was observed upon sunlight irradiation from 94% in *P. aeruginosa* to almost complete inactivation in *B. cereus*, which was attributed to the generated singlet oxygen from the dendrimer. A cotton fabric was treated with the dendrimer (DA15 and DA30), and its colorimetric characteristics were determined. Treatment of the fabric with the dendrimer partially increased its hydrophobicity and firmly attached the dendrimer to the surface, preventing its easy release into solution. Antimicrobial inactivation of the model bacterial strains in the presence of the cotton materials was tested in solution, in the dark, and after irradiation. In the dark, a weak inhibition of bacterial growth was observed, due to the fact that the dendrimer molecules are located only on the surface of the cotton matrix, without being released into solution, so their contact with microorganisms is limited to the fabric. After irradiation, a significant increase in antimicrobial activity was observed against both pathogenic strains, with the inactivation of *B. cereus* reaching 94% and of *P. aeruginosa* 89% (DA30 fabric), due to the singlet oxygen generated by the dendrimer. In this case, it is highly localized on the textile surface, enabling it to attack cell membranes and components of microorganisms effectively. This means that most bacterial inactivation is due to singlet oxygen. Scanning electron microscopy revealed no bacterial biofilm on the fabric surface. This photodynamic effect, combined with the fabric’s increased hydrophobicity, results in synergistic antibacterial and self-disinfecting properties that prevent bacterial cell adhesion and biofilm formation. The results demonstrate the potential of this new fluorescent dendrimer for antimicrobial photodynamic inactivation and the development of promising self-disinfecting textiles.

## Figures and Tables

**Table 1:** Photophysical properties of DA in different organic solvents.

Solvents	λ_A_ (nm)	λ_F_ (nm)	ν_A_–ν_F_ (cm^−1^)	ε (L mol^−1^ cm^−1^)	Φ_F_
Dimetyl sulfoxide	431	525	3996	81,950	0.36
*N*,*N*-dimnethylformamide	430	524	4008	87,010	0.38
Acetonitrile	426	515	4563	88,720	0.36
Methanol	432	531	4316	86,740	0.15
Ethanol	435	524	3905	86,330	0.11
Dichloromethane	415	515	4973	93,500	0.65
Dioxane	413	502	4293	81,080	0.81
Ethyl acetate	418	506	4161	84,060	0.40
Chloroform	412	513	4779	81,680	0.70
Tetrahydrofuran	422	508	4012	79,780	0.72

**Table 2:** Color characteristics of pristine cotton and fabrics DA15 and DA30 before and after washing.

Sample	L*	a*	b*	ΔL*	Δa*	Δb*	ΔE*
Pristine cotton	84.70	−0.10	0.56	-	-	-	-
Cotton + DA15 Before washing	81.73	−5.25	41.24	−2.97	−5.15	40.68	41.11
Cotton + DA15 After washing	82.13	−5.08	40.63	−2.57	−4.98	40.46	40.84
Cotton + DA30 Before washing	80.79	−3.75	43.52	−3.91	−3.65	42.96	43.29
Cotton + DA30 After washing	80.14	−3.59	42.82	−4.56	−3.49	42.65	42.98

## Data Availability

The original contributions presented in this study are included in the article/Supplementary Materials. Further inquiries can be directed to the corresponding authors.

## Supplementary Materials

The following supporting information can be downloaded at: https://www.mdpi.com/article/10.3390/ma18245570/s1: Methods, chemicals, and apparatus used in this work.

## References

[1] Sanders D., Grunden A., Dunn R.R. (2021). A review of clothing microbiology: The history of clothing and the role of microbes in textiles. Biol. Lett..

[2] Vojnits K., Mohseni M., Parvinzadeh Gashti M., Nadaraja A.V., Karimianghadim R., Crowther B., Field B., Golovin K., Pakpour S. (2024). Advancing Antimicrobial Textiles: A Comprehensive Study on Combating ESKAPE Pathogens and Ensuring User Safety. Materials.

[3] Gulati R., Sharma S., Sharma R.K. (2022). Antimicrobial textile: Recent developments and functional perspective. Polym. Bull..

[4] Roman L.E., Gomez E.D., Solis J.L., Gomez M.M. (2020). Antibacterial Cotton Fabric Functionalized with Copper Oxide Nanoparticles. Molecules.

[5] Staneva D., Atanasova D., Nenova A., Vasileva-Tonkova E., Grabchev I. (2021). Cotton Fabric Modified with a PAMAM Dendrimer with Encapsulated Copper Nanoparticles: Antimicrobial Activity. Materials.

[6] Mehravani B., Ribeiro A.I., Zille A. (2021). Gold Nanoparticles Synthesis and Antimicrobial Effect on Fibrous Materials. Nanomaterials.

[7] del Olmo N.S., Carloni R., Ortega P., García-Gallego S., de la Mata F.J. (2020). Metallodendrimers as a promising tool in the biomedical field: An overview. Adv. Organomet. Chem..

[8] Mahltig B., Tatlises B., Fahmi A., Haase H.J. (2013). Dendrimer stabilized silver particles for the antimicrobial finishing of textiles. J. Text. Inst..

[9] Jiao Y., Niu L.N., Ma S., Li J., Tay F.R., Chen J.H. (2017). Quaternary ammonium-based biomedical materials: State-of-the-art, toxicological aspects and antimicrobial resistance. Prog. Polym. Sci..

[10] Jennings M.C., Minbiole K.P., Wuest W.M. (2015). Quaternary Ammonium Compounds: An Antimicrobial Mainstay and Platform for Innovation to Address Bacterial Resistance. ACS Infect. Dis..

[11] Stan M.S., Chirila L., Popescu A., Radulescu D.M., Radulescu D.E., Dinischiotu A. (2019). Essential Oil Microcapsules Immobilized on Textiles and Certain Induced Effects. Materials.

[12] El-Tarabily K.A., El-Saadony M.T., Alagawany M., Arif M., Batiha G.E., Khafaga A.F., Elwan H.A.M., Elnesr S.S., El-Hack M.E.A. (2021). Using essential oils to overcome bacterial biofilm formation and their antimicrobial resistance. Saudi J. Biol. Sci..

[13] Falanga A., Del Genio V., Galdiero S. (2021). Peptides and Dendrimers: How to Combat Viral and Bacterial Infections. Pharmaceutics.

[14] Čuk N., Simončič B., Fink R., Tomšič B. (2024). Bacterial Adhesion to Natural and Synthetic Fibre-Forming Polymers: Influence of Material Properties. Polymers.

[15] Staneva D., Said A.I., Vasileva-Tonkova E., Grabchev I. (2022). Enhanced Photodynamic Efficacy Using 1,8-Naphthalimides: Potential Application in Antibacterial Photodynamic Therapy. Molecules.

[16] Staneva D., Vasileva-Tonkova E., Grabchev I. (2019). Chemical Modification of Cotton Fabric with 1,8-Naphthalimide for Use as Heterogeneous Sensor and Antibacterial Textile. J. Photochem. Photobiol. A Chem..

[17] Zhu H., Liu C., Su M., Rong X., Zhang Y., Wang X., Wang K., Li X., Yu Y., Zhang X. (2021). Recent advances in 4-hydroxy-1,8-naphthalimide-based small-molecule fluorescent probes. Coord. Chem. Rev..

[18] Wang Y., Li Y., Cao J., Yang X., Huang J., Huang M., Gu S. (2024). Research Progress of Fluorescent Probes for Detection of Glutathione (GSH): Fluorophore, Photophysical Properties, Biological Applications. Molecules.

[19] Gong H.H., Addla D., Lv J.S., Zhou C.H. (2016). Heterocyclic naphthalimides as new skeleton structure of compounds with increasingly expanding relational medicinal applications. Curr. Top. Med. Chem..

[20] Ruan W., Xie Z., Wang Y., Xia L., Guo Y., Qiao D. (2024). An Overview of Naphthylimide as Specific Scaffold for New Drug Discovery. Molecules.

[21] Alsaeedi H., Alabdullah I., Alsalme A., Khan R.A. (2026). Synthesis of 4-Chloro-1,8-Naphthalimide analogs: DNA binding, molecular docking, and in vitro cytotoxicity. J. Mol. Struct..

[22] Staneva D., Grabchev I., Kesharwani P. (2021). Chapter 20, Dendrimer as antimicrobial agents. Dendrimer-Based Nanotherapeutics.

[23] Jia J., Gao Y., Dang K., Guo X., Ding A. (2021). Naphthalimide-modified dendrimers as efficient and low cytotoxic nucleic acid delivery vectors. Polym. Int..

[24] Grabchev I., Angelova S., Staneva D. (2023). Yellow-Green and Blue Fluorescent 1,8-Naphthalimide-Based Chemosensors for Metal Cations. Inorganics.

[25] Said A.I., Staneva D., Grabchev I. (2023). New Water-Soluble Poly(propylene imine) Dendrimer Modified with 4-Sulfo-1,8-naphthalimide Units: Sensing Properties and Logic Gates Mimicking. Sensors.

[26] Said A.I., Staneva D., Vasileva-Tonkova E., Grozdanov P., Nikolova I., Stoyanova R., Jordanova A., Grabchev I. (2024). Synthesis, Spectral Characteristics, Sensing Properties and Microbiological Activity of New Water-Soluble 4-Sulfo-1,8-naphthalimides. Chemosensors.

[27] Cangiotti M., Staneva D., Ottaviani M.F., Vasileva-Tonkova E., Grabchev I. (2021). Synthesis and characterization of fluorescent PAMAM dendrimer modified with 1,8-naphthalimide units and its Cu(II) complex designed for specific biomedical application. J. Photochem. Photobiol. A Chem..

[28] Sadeghi-Kiakhani M., Safapour S. (2016). Functionalization of poly(amidoamine) dendrimer-based nano-architectures using a naphthalimide derivative and their fluorescent, dyeing and antimicrobial properties on wool fibers. Luminescence.

[29] Jordanova A., Tsanova A., Stoimenova E., Minkov I., Kostadinova A., Hazarosova R., Angelova R., Antonova K., Vitkova V., Staneva G. (2025). Molecular Mechanisms of Action of Dendrimers with Antibacterial Activities on Model Lipid Membranes. Polymers.

[30] (2024). WHO Bacterial Priority Pathogens List, 2024: Bacterial Pathogens of Public Health Importance to Guide Research, Development and Strategies to Prevent and Control Antimicrobial Resistance.

[31] Gauba A., Rahman K.M. (2023). Evaluation of Antibiotic Resistance Mechanisms in Gram-Negative Bacteria. Antibiotics.

[32] Uddin T.M., Chakraborty A.J., Khusro A., Zidan B.M.R.M., Mitra S., Emran T.B., Koirala N. (2021). Antibiotic resistance in microbes: History, mechanisms, therapeutic strategies and prospects. J. Infect. Public Health.

[33] Caivano G., Sciarra F.M., Messina P., Cumbo E.M., Caradonna L., Di Vita E., Nigliaccio S., Fontana D.A., Scardina A., Scardina G.A. (2025). Antimicrobial Resistance and Causal Relationship: A Complex Approach Between Medicine and Dentistry. Medicina.

[34] Coque T.M., Cantón R., Pérez-Cobas A.E., Fernández-de-Bobadilla M.D., Baquero F. (2023). Antimicrobial Resistance in the Global Health Network, Known Unknowns and Challenges for Efficient Responses in the 21st Century. Microorganisms.

[35] Irfan M., Almotiri A., AlZeyadi Z.A. (2022). Antimicrobial Resistance and Its Drivers—A Review. Antibiotics.

[36] Niño-Vega G.A., Ortiz-Ramírez J.A., López-Romero E. (2025). Novel Antibacterial Approaches and Therapeutic Strategies. Antibiotics.

[37] Salam M.A.A., Al-Amin M.Y., Salam M.T., Pawar J.S., Akhter N., Rabaan A.A., Alqumber M.A.A. (2023). Antimicrobial Resistance, A Growing Serious Threat for Global Public Health. Healthcare.

[38] Brüssow H. (2024). The Antibiotic Resistance Crisis and the Development of New Antibiotics. Microb. Biotechnol..

[39] Devi N.S., Mythili R., Cherian T., Dineshkumar R., Sivaraman G.K., Jayakumar R., Prathaban M., Duraimurugan M., Chandrasekar V., Peijnenburg W.J.G.M. (2024). Overview of antimicrobial resistance and mechanisms: The relative status of the past and current. Microbe.

[40] Gupta M., Sahu A., Mukherjee T., Mohanty S., Das P., Nayak N., Kumari S., Singh R.P., Pattnaik A. (2025). Divulging the potency of naturally derived photosensitizers in green PDT: An inclusive review of mechanisms, advantages, and future prospects. Photochem. Photobiol. Sci..

[41] Delcanale P., Abbruzzetti S., Viappiani C. (2022). Photodynamic treatment of pathogens. Riv. Nuovo Cim..

[42] Surur A.K., Barros de Oliveira A., De Annunzio S.R., Ferrisse T.M., Fontana C.R. (2024). Bacterial resistance to antimicrobial photodynamic therapy: A critical update. J. Photochem. Photobiol. B.

[43] Amendola G., Di Luca M., Sgarbossa A. (2025). Natural Biomolecules and Light: Antimicrobial Photodynamic Strategies in the Fight Against Antibiotic Resistance. Int. J. Mol. Sci..

[44] Polat E., Kang K. (2021). Natural Photosensitizers in Antimicrobial Photodynamic Therapy. Biomedicines.

[45] Aires-Fernandes M., Botelho Costa R., Rochetti do Amaral S., Mussagy C.U., Santos-Ebinuma V.C., Primo F.L. (2022). Development of Biotechnological Photosensitizers for Photodynamic Therapy: Cancer Research and Treatment—From Benchtop to Clinical Practice. Molecules.

[46] Correia J.H., Rodrigues J.A., Pimenta S., Dong T., Yang Z. (2021). Photodynamic Therapy Review: Principles, Photosensitizers, Applications, and Future Directions. Pharmaceutics.

[47] Sai D.L., Lee J., Nguyen D.L., Kim Y.P. (2021). Tailoring photosensitive ROS for advanced photodynamic therapy. Exp. Mol. Med..

[48] Wang T.Y., Libardo M.D.J., Angeles-Boza A.M., Pellois J.P. (2017). Membrane Oxidation in Cell Delivery and Cell Killing Applications. ACS Chem. Biol..

[49] Staneva D., Vasileva-Tonkova E., Grozdanov P., Vilhelmova-Ilieva N., Nikolova I., Grabchev I. (2020). Synthesis and photophysical characterisation of 3-bromo-4-dimethylamino-1,8-naphthalimides and their evaluation as agents for antibacterial photodynamic therapy. J. Photochem. Photobiol. A Chem..

[50] Manov H., Staneva D., Vasileva-Tonkova E., Grozdanov P., Nikolova I., Stoyanov S., Grabchev I. (2021). Photosensitive dendrimers as a good alternative to antimicrobial photodynamic therapy of Gram-negative bacteria. J. Photochem. Photobiol. A Chem..

[51] Grabchev I., Jordanova A., Vasileva-Tonkova E., Minkov I.L. (2024). Sensing and Microbiological Activity of a New Blue Fluorescence Polyamidoamine Dendrimer Modified with 1,8-Naphthalimide Units. Molecules.

[52] Gong Z.-L., Pan Q.-J., Ma D.-X., Zhong Y.-W. (2023). Naphthalimide-Modified Tridentate Platinum(II) Complexes: Synthesis, Characterization, and Application in Singlet Oxygen Generation. Inorganics.

[53] Magri D.C., Johnson A.D. (2025). Naphthalimide–organometallic hybrids as multi-targeted anticancer and luminescent cellular imaging agents. RSC Med. Chem..

[54] Luo X., Yang Y., Qian X. (2020). Recent progress on the development of thioxo-naphthalimides. Chin. Chem. Lett..

[55] Staneva D., Angelova S., Grabchev I. (2020). Spectral Characteristics and Sensor Ability of a New 1,8-Naphthalimide and Its Copolymer with Styrene. Sensors.

[56] Orasugh J.T., Temane L.T., Pillai S.K., Ray S.S. (2025). Advancements in Antimicrobial Textiles: Fabrication, Mechanisms of Action, and Applications. ACS Omega.

[57] Vaideki K., Jayakumar S., Rajendran R., Thilagavathi G. (2008). Investigation on the effect of RF plasma and neem leaf extract treatment on the surface modification and antimicrobial activity of cotton fabric. Appl. Surf. Sci..

[58] Mosinger J., Mosinger B. (1995). Photodynamic sensitizers assay: Rapid and sensitive iodometric measurement. Experientia.

[59] Wan L., Yiming X. (2013). Iodine-sensitized oxidation of ferrous ions under UV and visible light: The influencing factors and reaction mechanism. Photochem. Photobiol. Sci..

[60] Glaeser J., Nuss A.M., Berghoff B.A., Klug G. (2011). Singlet Oxygen Stress in Microorganisms. Adv. Microb. Physiol..

[61] Maisch T., Baier J., Franz B., Maier M., Landthaler M., Szeimies R.M., Bäumler W. (2007). The role of singlet oxygen and oxygen concentration in photodynamic inactivation of bacteria. Proc. Natl. Acad. Sci. USA.

