# Supplementary material for: Photodynamic Microbial Defense of Cotton Fabric with 4-Amino-1,8-naphthalimide-Labeled PAMAM Dendrimer
Source: Materials (Basel). 2025 Dec 11;18(24):5570. doi: 10.3390/ma18245570 (PMC12734544; doi:10.3390/ma18245570)
Supplement: Supplementary file 1 [file materials-18-05570-s001.zip › materials-4024053-supplementary.pdf]

Supplementary Materials

Photodynamic microbial defence of cotton fabric with amino-1,8-naphthalimide labelled PAMAM dendrimer

Desislava Staneva[1,*], Daniela Atanasova[1], Ivo Grabchev[2*]

[1]*University of Chemical Technology and Metallurgy, 1797 Sofia, Bulgaria*

[2.]*Sofia University "St. Kliment Ohridski", Faculty of Medicine, 1407 Sofia, Bulgaria*

*Correspondence: grabcheva@mail.bg (D.S.); i.grabchev@chem.uni-sofia.bg (I.G.)

Materials and Methods

The photophysical characteristics of dendrimer in organic solvents has been determinate at Thermo Spectronic Unicam UV 500 UV-Vis spectrophotometer and the Cary Eclipse spectrofluorometer (Varian, Austria). Organic solvents (ACN - Acetonitrile; CHCl3 - Chloroform; 1,4-Dioxane; DCM - Dichlormethane; DMSO - Dimethyl sulfoxide; DMF - *N,N*-Dimethylformamide; EtOH - Ethanol; EtOAc - Ethyl acetate; MeOH - Methanol) was of spectroscopic grade and has been used as obtained from (Sigma Aldrich, Germany). Absorption and fluorescence spectra were recorded using synthetic quartz glass cells with a 1 cm path length, at a dendrimer concentration of 10^{-6} mol L^{-1}. The progress of the condensation reaction was monitored by thin-layer chromatography on silica gel (Fluka F60 254, 20x20; 0.2 mm), utilizing a hexane/acetone (2:1) system as the elution phase. NMR spectra were recorded on a Bruker Avance III HD spectrometer with working frequencies of 500.13 MHz for the protons and 125.8 MHz for 13C and temperature 298.0 ± 0.1K. A Fourier-transform spectrometer (IRAffinity-1 Shimadzu) equipped with a diffuse-reflectance attachment (MIRacle Attenuated Total Reflectance Attachment) was used at a resolution of 1 cm^{-1}. A Jeol JSM-5510 scanning electron microscope was used for SEM characterization of the samples.

1. *Iodometric measurements*

To an aqueous solution of KI (20 mL, 0.5 M), $1x10^{-6}$ M dendrimer DA, or cotton fabrics treated with (size of 1 cm^2), are added and irradiated with a Newport solar simulator (150 W Xe, 36 mW/cm^2) for 60 minutes. The absorption spectra of the solution are recorded at intervals of 5 minutes. The distance between the simulator and the samples was 25 cm.

2. *Cotton fabric treated with dendrimer*

The 100% cotton fabrics were treated with a dendrimer DA solution in ethanol at concentrations of 0.15% and 0.30% based on the weight of the fabric. The ratio of ethanol to fabric was set at 30:1, and the treatment was conducted at a temperature of 50°C for 60 minutes. After treatment, the fabrics were removed and allowed to air dry at room temperature. They were then rinsed three times with water and a detergent solution (Lavoral S 313; Bozzeto group) at a concentration of 2 g L^{-1}, followed by another air drying process. The resulting textile fabrics were subsequently used for further studies.

3. *In vitro antimicrobial tests*

The antimicrobial activity of the dendrimer DA was tested against Gram-positive *Bacillus cereus* and Gram-negative *Pseudomonas aeruginosa* as model bacterial strains. Its ability to inhibit the growth of the model pathogens was tested in meat-peptone broth (MPB) in dark and under light illumination. The dendrimer was dissolved in DMSO at a started concentration of 1.0 mg mL^{-1} and further diluted in test tubes with meat-peptone broth (MPB) to final concentrations of 50, 40, 20 and 10 μg mL^{-1}. Two sets of tubes were prepared for the experiments in presence and absence of light. After inoculation with each standardized cell suspension, the tubes were incubated at appropriate temperature for 18 h under shaking (at 240 rpm). Positive controls (compound and MPB, without inoculum) and negative controls (MPB and inoculum, without compounds) were used. The optical density of the medium at 600 nm (OD_{600}) was determined as a measure of microbial growth. The experiments were conducted in triplicate and the averages were taken (standard deviations less than 5%).

4. *Antimicrobial assay of the treated cotton fabrics*

The antimicrobial activity of DA-treated cotton fabric was tested in MPB against the model strains *Bacillus cereus* and *Pseudomonas aeruginosa* under light irradiation and in the dark. Tubes containing MPB and square-shaped cotton samples (10 mm x 10 mm) were inoculated with each microbial suspension. Tubes with natural cotton and no samples were also prepared as controls. Two sets of tubes were ready for testing in the presence and absence of light. After 18 hours of incubation at the appropriate temperature, the samples were removed, and the OD_{600} was determined. The antimicrobial activity of the samples was assessed by the reduction of bacterial growth in the presence of the treated samples compared to the native untreated samples. The tests were performed in triplicate, and the mean values (standard deviations < 5%) are reported.
